# Morphology and Luminescence of Flexible Free-Standing ZnO/Zn Composite Films Grown by Vapor Transport Synthesis

**DOI:** 10.3390/ma15228165

**Published:** 2022-11-17

**Authors:** Ludmila A. Zadorozhnaya, Andrey P. Tarasov, Ivan S. Volchkov, Arsen E. Muslimov, Vladimir M. Kanevsky

**Affiliations:** Shubnikov Institute of Crystallography, Federal Scientific Research Centre “Crystallography and Photonics” of Russian Academy of Sciences, 119333 Moscow, Russia

**Keywords:** ZnO, free-standing film, zinc film, Si whiskers, VLS growth, flexible film, highly developed surface, UV luminescence, deep-level emission suppression

## Abstract

A method for fabricating flexible free-standing ZnO/Zn composite films from the vapor phase using a regular array of silicon microwhiskers as a substrate is presented. The structural and morphological peculiarities, as well as luminescent properties of the films, were studied. The films have a hybrid structure consisting of two main microlayers. The first layer is formed directly on the tops of Si whiskers and has a thickness up to 10 µm. This layer features a polycrystalline structure and well-developed surface morphology. The second layer, which makes up the front side of the films, is up to 100 µm thick and consists of large microcrystals. The films show good bending strength—in particular, resistance to repeated bending and twisting—which is provided by a zinc metallic part constituting the flexible carrier of the films. ZnO photoluminescence was observed from both surfaces of the films but with conspicuous spectral differences. In particular, a significant weakening of ZnO green luminescence (more than 10 times) at an almost constant intensity of UV near-band edge emission was found for the polycrystalline side of the films as compared to the microcrystalline side. A high degree of homogeneity of the luminescent properties of the films over their area was demonstrated. The results obtained emphasize the relevance of further studies of such ZnO structures—in particular, for application in flexible devices, sensors, photocatalysis and light generation.

## 1. Introduction

Semiconductor planar technologies are a well-developed field in modern science and engineering, covering a huge number of research trends related to optical and optoelectronic technologies, integrated circuits, sensors, flexible electronics, biological and chemical technologies and having a lot of applications [1,2,3,4]. One of the promising building blocks of such technologies are free-standing films, which have gained interest due to their special properties—particularly, the absence of mechanical stresses due to contact with the substrate—and the possibility in using them for different applications, including membranes, filters, flexible electronics, photocatalysts and catalyst carriers, bioactive coatings and other devices characterized, among others, by light weight and flexibility [5,6,7,8].

Due to their properties, nano- and microstructures, including films of ZnO, which is a direct-gap semiconductor with a wide bandgap (3.3–3.4 eV at room temperature), satisfy many practical demands—in particular, for applications in optoelectronics, spintronics, piezoelectric devices, chemical and biological sensors [9,10,11,12,13]—and have prospects for the expansion of their functionality [14,15,16,17,18,19]. A wide range of techniques can be applied to grow ZnO films, including physical ones, such as magnetron sputtering, pulsed laser deposition and molecular beam epitaxy [9,10,15,16], and chemical ones, e.g., chemical vapor deposition, sol–gel method, spray pyrolysis and spin coating [11,18,19]. Several methods can be used for the fabrication of free-standing ZnO films, as well. Among them are synthesis in an aqueous solution and at the liquid–air interface, sol–gel and hydrothermal methods [20,21,22,23].

A crucial task in obtaining free-standing films and similar substrate-free structures is related to enhancing their mechanical properties—in particular, increasing the tensile strength and resistance to various deformations [24,25,26,27,28]. Films and many types of ZnO nanostructures, which are quite brittle without substrates, are no exception. Not least for this reason, composite films based on ZnO are often made to obtain flexible properties, for example, by growing ZnO on flexible polymer substrates or matrices [29,30,31].

In the present work, a method for obtaining flexible free-standing ZnO/Zn composite films using the gas transport method within a single synthesis experiment was demonstrated. The possibility of obtaining such films due to their growth on silicon substrates with a preliminarily formed system of vertically aligned silicon microwhiskers was shown. The films were studied by electron and atomic force microscopy, energy-dispersive microanalysis, X-ray diffraction analysis and photoluminescent spectroscopy in various excitation regimes. The presence of a zinc metal phase provided the films with high flexibility and tensile strength. ZnO luminescence was observed from both sides of the films. At the same time, a strong suppression of ZnO visible luminescence on the substrate side was revealed. A high degree of homogeneity of the radiative properties of the films over their area was demonstrated.

## 2. Materials and Methods

**Figure 1:**
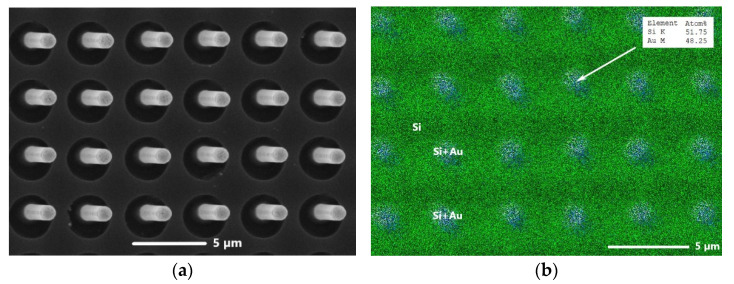
Electron microscopy image (**a**) and element mapping image (**b**) of a silicon substrate with a regular system of Si whiskers of the same height with Si-Au globules at the tops (view at an angle of 30° from the normal to the substrate surface). The inset in (**b**) shows the Au and Si contents in at.% at the top of one of the Si whiskers.

ZnO samples were synthesized by the gas transport method from zinc and oxygen vapors according to the vapor–liquid–solid (VLS) mechanism, in which the liquid phase plays the catalytic role. As substrates, we used (111)-oriented silicon wafers with vertically aligned silicon whiskers formed on the wafers, according to the procedure described in [32]. The whiskers were arranged periodically on the substrate with an interval of 5 μm, determined by a photolithography mask. During the growth of Si whiskers, a thin gold layer (~100 nm) was used as a catalyst, which formed a fusible eutectic with silicon. The technique used [32] ensures the preservation of the Si-Au eutectic alloy only at the tops of Si whiskers in the form of globules. A typical picture of a regular system of silicon whiskers with Si-Au globules at the tops is shown in Figure 1a,b (electron microscopy image and element mapping image).

Before the process of ZnO crystallization, the substrates were heated to a temperature of 600 °C. Next, the temperatures in the evaporation and growth zones were gradually brought up to the operating value (660 and 580 °C, respectively). After that, oxygen of special purity grade was supplied to the reactor. The synthesis time was 30 min. A detailed description of the synthesis process and related equipment was presented in [33]. Several ZnO films were fabricated during the present research. For comparison purposes, we present results for two of the films synthesized in different experiments under nominally identical conditions, using Si substrates 10 × 10 mm in size (films N1 and N2).

Microscopic studies of the samples were carried out via scanning electron microscopy (SEM), using a Jeol JSM-6000PLUS microscope equipped with an energy-dispersive X-ray (EDX) microanalyzer and atomic force microscopy (AFM), using a NT-MDT Solver PRO M scanning probe microscope. The crystal structure of the samples was studied by X-ray diffraction (XRD) analysis using a Miniflex 600 diffractometer equipped with a diffracted beam monochromator set for CuK_α_ radiation (λ = 1.54056 Å) at an accelerating voltage of 40 kV and a current of 15 mA. The measurements were carried out in Bragg–Brentano geometry in the *θ*–2*θ* scanning mode. The 2*θ* scan range was 30–80°, with a step size of 0.01° and a step time of 1.0 s.

The photoluminescence (PL) of the structure was studied at room temperature (RT) using low-intensity *cw* excitation with a xenon lamp and relatively intensive optical excitation with a pulsed laser source. For low-intensity excitation measurements, a Varian Cary Eclipse spectrofluorometer equipped with a xenon lamp was used. In this case, radiation with a wavelength of 315 nm was used to excite the PL of the samples. High-intensity excitation was carried out using the 3rd harmonic of a Nd:YAG laser, providing pulsed radiation with a wavelength of 355 nm, a repetition rate of 15 Hz and a pulse duration of 10 ns. The excitation power density used in this case was 40 kW/cm^2^.

## 3. Results

**Figure 2:**
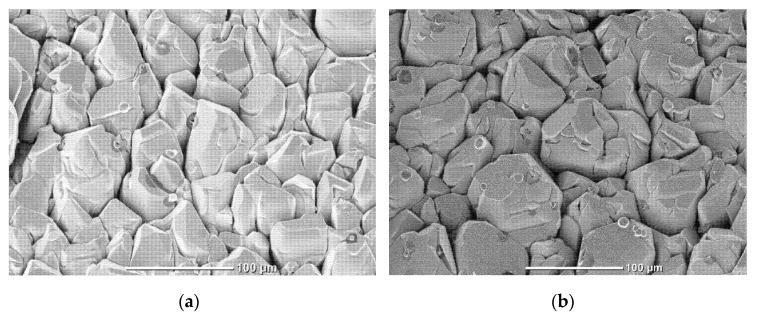
Planar SEM images of studied ZnO films (front side): (**a**) N1 film and (**b**) N2 film.

SEM images of the obtained ZnO films are shown in Figure 2a,b (N1 and N2). The films consist of microcrystallites up to ~100 μm in size, with an average size of ~30 μm. These microcrystallites do not form close packing; there are gaps between them up to 5 µm in size.

**Figure 3:**
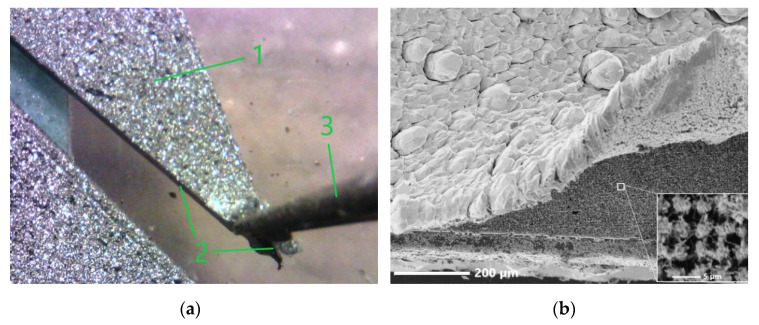
Obtaining free-standing films: (**a**) trial mechanical detachment of one of the fabricated films (1) from the substrate (2) using tweezers (3) (a photograph taken with an optical digital microscope; the size of the displayed area in the plane of the sample surface is ~5.1 × 3.8 mm). (**b**) The edge of the film N1 peeled off the substrate (a SEM image); the inset shows the substrate area with the system of Si whiskers after peeling off the film.

In the course of the study, it was found that the fabricated films can be rather easily isolated from the substrates without destruction. Figure 3a shows a photograph of the process of peeling one of the fabricated films off the substrate, taken with an optical digital microscope. At the same time, for a semiconductor material, especially microfilms, such films demonstrated an amazing degree of resistance to tearing and plastic deformation; not only were they not destroyed during mechanical detachment from the substrate, but they also withstood repeated episodes of bending and twisting.

Figure 3b shows the SEM image of the N1 film’s edge at the place of its separation from the substrate. It can be seen that the microcrystalline part of the film is a monolayer of microcrystallites. Undamaged Si whiskers are visible under the film. Near their tops, filament-like ZnO nanocrystals are observed in the form of nanobushes and nanowhiskers (see inset in Figure 3b).

**Figure 4:**
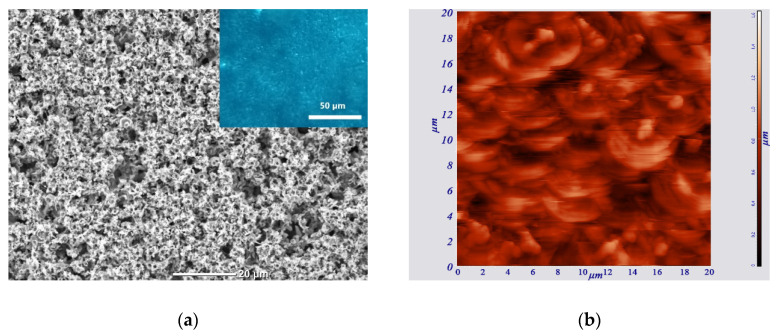
Planar images of the reverse side of the N1 film, obtained using: (**a**) SEM (the inset shows an optical microscopy image obtained in the transmission mode) and (**b**) AFM.

Already during the process of detachment of the films from the substrates, it was noticed that the appearance of their reverse side, which was in contact with the substrate, differs from that of the front side. Particularly, the front side surface was visually brighter and had a greater degree of light scattering than the back surface. In the SEM image shown in Figure 3b, where the surface of the reverse side of the N1 film is at an angle for the observer, it can be seen that the relief of this part is characterized by a smaller height difference compared to the front side of the film. A more detailed study of the reverse side of the films revealed its peculiarities. In particular, in Figure 4a, which shows a planar SEM image of the reverse side of the N1 film, one can observe the well-developed surface morphology. Optical microscopy revealed the rare presence of pores up to several microns in size in some areas of the film (see the inset in Figure 4a).

Figure 4b shows the AFM image of the surface of the film’s reverse side. Since the back surface of the films had a developed polycrystalline structure, the contact mode was used, which made it possible to display both the topography and the slightest relief differences. The data obtained made it possible to clarify in more detail the morphology of the polycrystalline structure. In particular, this structure is formed by fairly large microcrystals up to 4 µm in size, which is believed to be zinc, as well as agglomerates of small, presumably ZnO, nanocrystallites up to 250 nm in size. The root mean square surface of the microcrystals’ roughness is 15.6 nm, and the average size of the individual small nanocrystallites was 50–100 nm.

**Figure 5:**
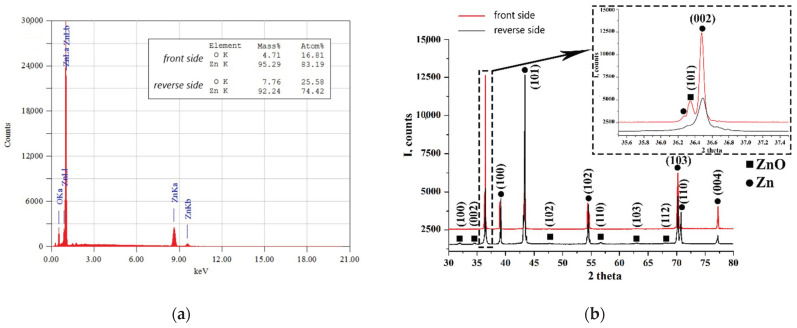
Structural and element comparison of the front and reverse sides of the N1 film: (**a**) a typical EDX spectrum of the film (the front surface); the inset shows the composition of elements at particular areas of both surfaces of the film. (**b**) XRD patterns for the front (top, red line) and reverse (bottom, black line) sides of the film; squares and circles indicate ZnO and Zn reflections, respectively.

To further compare the front and reverse sides of the films, EDX, XRD and PL measurements were performed. Figure 5a shows a typical EDX spectrum of the films under study by the example of the N1 film’s front surface. The inset in Figure 5a compares the contents of the elements measured at particular areas of the front and back surfaces of the N1 film. In general, this analysis confirmed that the zinc content was higher than the oxygen content over the entire area of both sides of the films. At the same time, the Zn/O ratio averaged over the sample’s surface was higher for the front side than for the reverse side. In particular, the averaged Zn/O ratio was 82/18 in terms of at.% in the case of the front surface and 74/26 at.% in the case of the back surface. Of note is that no traces of gold or silicon were detected in the films by the EDX analysis. Taking into account the large thickness of the films, the rather long synthesis, and the small thickness of the gold layer, it can be assumed that the Si-Au eutectic compound either entered the film structure or evaporated during the synthesis.

Figure 5b shows XRD patterns obtained from the front and reverse sides of the N1 film. The presence of a ZnO (JCPDS pattern for ZnO no: 043-0002) wurtzite structure and Zn phase (JCPDS pattern for Zn no: 001-1244) was observed when scanning from both sides. However, the polycrystalline phase of ZnO was more pronounced when scanning from the reverse side of the film. The coherent scattering regions (crystallite sizes) calculated from the XRD data are approximately 25 and 73 nm, respectively. The ZnO cell parameter is reduced in comparison with the standard parameter, which indicates a lack of oxygen. In addition, one main reflection from ZnO (101) is clearly visible on the front side of the film (see the inset in Figure 5b), while low-intensity peaks of various crystallographic orientations are observed on the reverse side: (100), (002), (101), (102), etc. This indicates the polycrystalline nature of the film’s reverse side and dominant (101) orientation of the ZnO microstructure on the front side. It is also worth noting that the prevailing orientation of the zinc phase also differs between the film’s sides. In particular, while on the reverse side, Zn crystallites are predominantly (101)-oriented, they exhibit prevailing (002) orientation on the front side.

**Figure 6:**
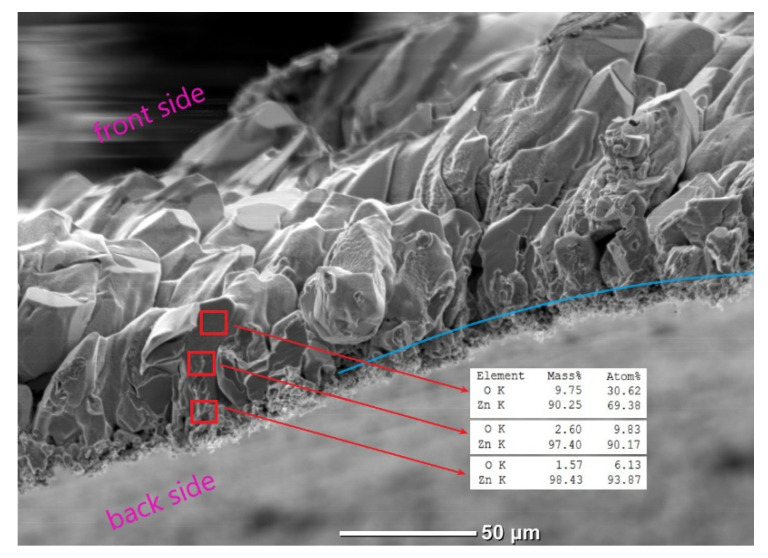
SEM image of the rupture region of the N1 film. Compositions of elements are shown for the areas marked with red squares. The blue line shows the approximate position of the boundary between the microcrystalline and nanocrystalline parts of the film.

To clarify the composition of the films alongside their depth—in particular, to establish the localization of the zinc phase—SEM and EDX studies of the rupture and slice regions of the films were carried out. First of all, these studies revealed the presence of a sufficiently clear boundary between their microcrystalline and polycrystalline parts. Figure 6 shows the SEM image of the N1 film’s rupture region, and the EDX microanalysis results in three areas of this region located at different depths of the film. It can be seen that the film thickness is approximately composed of the microcrystals’ size on the front part of the film and the thickness of the polycrystalline layer on the reverse side, which is up to 10 µm. The EDX analysis reveals a different ratio of zinc and oxygen alongside the film’s depth—particularly, a monotonic decrease in the zinc content in the direction from the back surface to the front surface of the film. In particular, the Zn/O ratio reaches ~94/6 in terms of at.% in the back part of the film and drops down to ~69/31 at.% in the near-surface region of the front part.

**Figure 7:**
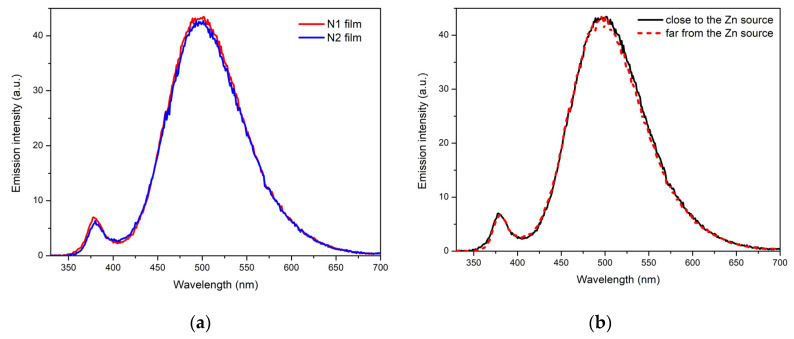
PL spectra of the front surfaces of the films under low-intensity excitation: (**a**) N1 (red) and N2 (blue) films. (**b**) Two opposite parts of the N1 film located at different distances from the Zn vapor source during the ZnO synthesis. The black solid (red dashed) line is for the sample’s area closest to (farthest from) the Zn source.

Figure 7a shows the PL spectra of the front sides of the films (approximate centers of the samples) were recorded at a low-intensity excitation using an Xe lamp. The spectra consist of UV and visible components with maxima at 379 nm (3.27 eV) and 496 nm (2.5 eV), respectively. The UV component is the near-band edge (NBE) luminescence of ZnO. Its full width at half-maximum (FWHM) is 22 nm. This value is slightly increased due to partial overlap with the visible component. Taking the band gap energy of ZnO as 3.37 eV at RT [34], we can assume that the edge component is a mixture of free exciton (FX) emission (exciton binding energy in ZnO is 60 meV) and its first phonon replica (FX-LO), which is spaced from FX by about 50–60 meV at RT [35,36]. Visible luminescence is often referred to as the deep-level emission (DLE) of ZnO and is associated with radiative transitions involving the deep energy levels of intrinsic defects.

Despite the slightly different microstructures of the films, we can speak of their similar radiant intensity on the microscale (the transverse size of the excitation spot on the sample is 1 mm), since their PL spectra exhibit the same shape and only slight differences in intensity. The integral intensities over the entire spectrum differ between the films by 2%, while, in the case of the UV component, this ratio is ~15% higher for the N1 film. This indicates a good reproducibility of the synthesis of such structures.

To check the uniformity of the optical properties of the films fabricated, we studied luminescence in different parts of the films. Figure 7b shows the PL spectra of the front side of N1 film recorded at a low excitation intensity in two opposite parts of the sample, corresponding to different distances from the zinc source during synthesis. It can be seen that the spectra are almost completely identical, which indicates good homogeneity (on the microscale) of the film.

**Figure 8:**
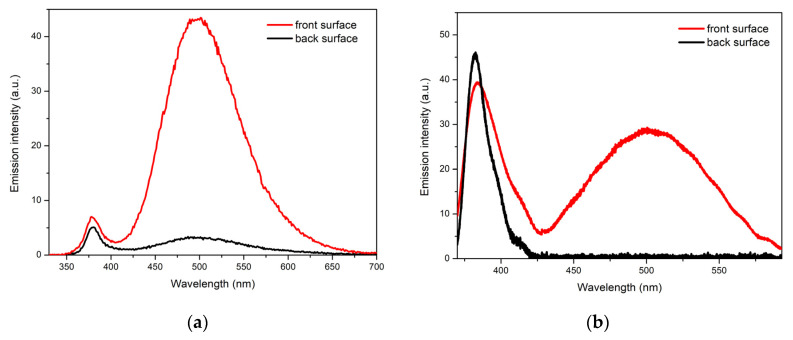
PL spectra of the front (red) and back (black) surfaces of the N1 film under: (**a**) low-intensity excitation and (**b**) pulsed laser excitation.

When studying the PL properties of the films, an interesting feature was revealed: a strong difference in the PL character for the front and back parts of the films. Figure 8a compares the PL spectra of both sides of the N1 film as an example. In this case, one can notice a significant difference in the ratios of the DLE and NBE bands from different sides of the film at the same spectral positions of the bands. In particular, while, for the front side, this ratio is equal to 27 in terms of integral intensities and 6 in terms of the maxima, for the reverse side, these values are approximately 3.5 and 0.5, respectively. Thus, the DLE/NBE ratio is approximately an order of magnitude lower for the reverse side of the film. Of note, the DLE intensity between both sides differs by a factor of ~11 in terms of integral intensities and 14 times in terms of the maxima. The difference in the intensities of the NBE component is about 25% in favor of the front side. A similar picture was observed in the case of the N2 film. Thus, a significant DLE suppression is observed for the reverse side of the films at a much smaller change in the UV signal.

The PL of the N1 sample on both sides was also studied under relatively intensive excitation using a pulsed laser source (Figure 8b). NBE emission, in this case, is represented by a band with a maximum at 382–383 nm. The visible emission is completely absent in the PL spectrum of the reverse side of the film. The DLE band with a maximum at about 500 nm is observed only on the front surface. The DLE/NBE ratio in this case constitutes ~2 (in terms of the integrated intensities), i.e., more than 10 times lower compared to the case of low-intensity excitation.

In the case of pulse laser excitation, the local inhomogeneity of the sample plays a more important role as compared to low-intensity *cw* photoexcitation, since the area of the excitation spot (~200 μm) is of the same order as the dimensions of the individual microcrystallites. In view of this, the radiation intensity, the position and width of the NBE emission band, as well as the ratio of the UV and visible emission components, may vary slightly from place to place (especially on the front surface of the film). However, in general, the effect of a significant decrease in the DLE intensity on the reverse side of the film (down to zero) is also observed in this case.

## 4. Discussion

Gold forms a fusible eutectic with silicon; as a consequence, at the crystallization temperature of zinc oxide, the globule at the top of the whisker is a melt of silicon in gold. The EDX analysis of the Si whisker’s top revealed a Si-to-Au ratio content of nearly 50/50 in terms of at.% (see Figure 1b). The surface of a quasi-liquid globule can be considered ideally rough; due to which, the barrier to the substance condensation and the creation of solution supersaturation is reduced [32,37,38]. It is important that ZnO grows only in the area seeded by the Si + Au liquid phase. Thus, the films were formed directly on the tops of Si whiskers. The small contact area of the film with the substrate made it easy to separate them from each other and, ultimately, to obtain a free-standing film.

Synthesis under conditions of a significant excess of zinc resulted in the formation of: (i) ZnO filamentary nanocrystals on the tops of Si whiskers due to the appearance of numerous nucleation centers [38,39,40] and (ii) the metallic zinc phase during synthesis due to a significant increase of the mass of excess zinc on the mass of zinc that is involved in the reaction with oxygen [41].

**Figure 9:**
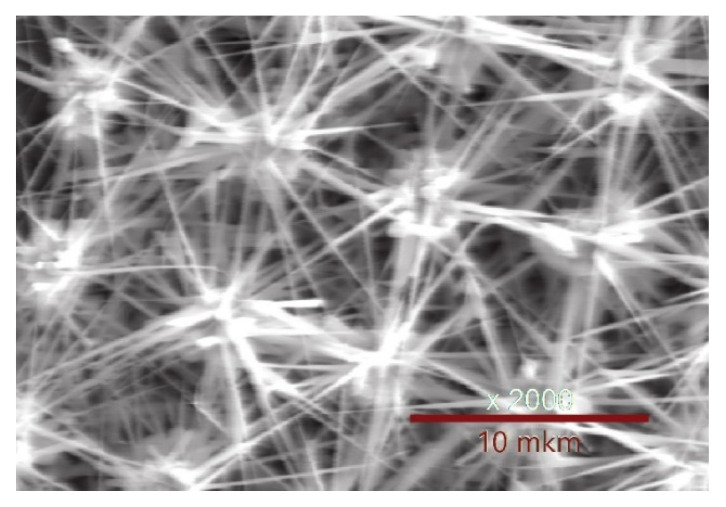
SEM image of ZnO nanowires on Si whiskers.

The formation of ZnO nanowires can be seen in Figure 9, showing a micrograph of a ZnO nanostructure grown by a similar technique on silicon substrates with whiskers. In this case, the distance between adjacent Si whiskers was ~10 μm, and the growth time was 10 min. One can see that the growth of ZnO nanowires and nanoneedles was seeded and catalyzed only on Au-Si globules at the tops of Si whiskers. In this case, a sufficiently large distance between the whiskers allows nanowires to reach 10 μm or more in length already for a short synthesis time, which is ensured by fast-catalyzed growth.

In the present study, similar ZnO filamentary nanocrystals on the tops of Si whiskers with Si-Au globules were formed at the first stage of synthesis. This was followed by the formation of the ZnO/Zn polycrystalline layer, which is confirmed by the EDX, XRD and AFM data for the back surface. This layer is a kind of buffer layer between the regular system of Si whiskers and microcrystalline part of the films and forms a well-developed back surface of the films. As the synthesis continues, due to a significant excess of zinc, the crystallinity of the zinc phase rises, and zinc crystallites with a certain prevailing orientation begin to appear. These crystallites increase in size and coalesce, forming both individual microdroplets and a zinc film structure. The resulting zinc phase served as seeds and catalysts for further ZnO growth, according to the “self-catalyzed” VLS mechanism [41].

By controlling the oxygen flow and, thus, the amount of reacting zinc, it is possible to control the ZnO/Zn ratio and, in particular, the degree of formation of a metal film, from its complete absence to a continuous layer throughout the intermediate stage of individual microcrystals and/or a porous structure. In the present study, individual micropores observed in some areas of the films indicate the implementation of an intermediate stage when a porous zinc layer is formed. Nevertheless, this layer is rather close to a continuous one and eventually gives a flexibility property for the peeled-off films.

Another extreme example, when the zinc phase is completely absent, is shown in our work [40], where we also studied the growth of ZnO on substrates with a regular system of Si whiskers with Au-Si globules using the vapor transport method. In [40], due to low supersaturations of zinc during synthesis, in a ZnO structure with the periodicity of the regular Si whisker system, every single ZnO microcrystal was formed on one Si whisker. The complete transparency of the crystals in visible light confirmed the absence of a metallic zinc phase. At the same time, in [40], a PL signal of the same order of magnitude as for the films currently under study, along with the noticeable predominance of DLE, also peaking in a green spectral region, over NBE emission, was observed. The possibility of forming sufficiently thick ZnO layers of micron thickness on Zn droplets is confirmed by laser studies of such structures. In particular, in [42], stimulated emission and whispering gallery mode lasing were observed in isometric ZnO microcrystals up to 80 µm in size, presumably formed from zinc microdrops. As is known, the optical gain, which provides lasing of this type, is formed in the near-surface region of the crystals [43]. Based on this, it can be assumed that the thickness of the ZnO layer covering the microcrystalline part of the films is on the order of micrometers.

At the same time, at first glance, a certain contradiction can be noted when comparing the EDX data for the film’s front surface and the rupture region. In particular, the increase of the zinc content on the front surface as compared to the contiguous near-surface region is observed. This can be due to turning off the oxygen supply to the growth chamber at the last stage of the synthesis. In this case, the argon flow continues and carries the zinc residues from the zinc source to the growth zone. A greater excess of zinc on the front surface compared to the back nanocrystalline part of the films causes a greater oxygen deficiency, and, consequently, the formation of a greater number of oxygen vacancies. This is reflected in the PL spectra of the films. In particular, compared to the films’ back surface, their front surfaces show a more intensive DLE band, peaking in the green spectral region (2.5 eV), which is usually associated with oxygen vacancies in ZnO [44,45].

Thus, the films under study exhibit radically different UV/visible luminescence ratios, depending on their sides. It can be assumed that this ratio, and hence, the content of defects, can be additionally controlled using post-growth processing, for example, annealing in O_2_ or Ar ambient [46,47]. These features can be useful when developing, on the one hand, UV radiation sources, where visible luminescence is an obstacle, and on the other hand, photocatalytic devices, since the defects responsible for DLE ensure the capture of free carriers produced by photoexcitation and delay inter-band recombination, which, in turn, contributes to an increase in the photocatalytic activity [48,49].

The phenomenon of the relative weakening of the DLE band with an increase in the photoexcitation intensity, observed in our experiment (Figure 8), was mentioned also in earlier studies of various ZnO micro- and nanostructures [44,50,51]. In particular, it is most pronounced in a situation such as ours, when the transition from weak *cw* excitation to relatively intensive pulsed excitation is performed [40,52,53]. This phenomenon can be associated with the effects of saturation of DLE centers and straightening of energy bands, which take place during the high-power excitation of a semiconductor [50,54]. In the second case, electron–hole pairs, intensively created by photons with an energy greater than the band gap, neutralize the excess charge on the semiconductor surface, the existence of which, in the absence of illumination, causes bending of the energy bands and formation of the depletion layer. The straightening of the bands is accompanied by a partial deactivation of DLE centers located near the surface and an increase in the probability of inter-band transitions. All this results in a relative increase in the UV component as compared to the visible one.

For many practical purposes, a sufficient degree of homogeneity of the fabricated samples is required. This is also often important when studying their physical—in particular, optical—properties. At the same time, using the vapor transport method and the VLS mechanism for ZnO growth do not always ensure the uniformity in the formation of ZnO crystals: the morphology and crystalline quality of the obtained structure may differ in different parts of the substrate. As a consequence, the physical—in particular, optical properties within the structure, e.g., an array of micro/nanocrystals—also differ. For example, in [55,56], where ZnO structures synthesized from the vapor phase were studied, it was shown that the ratio of the NBE and DLE spectral bands can differ significantly for the crystals formed at different distances from the source of Zn vapor.

A high degree of homogeneity of the films studied in the present work was confirmed by a constant level of the luminescence intensity (both in the UV and visible regions of the spectrum) in the different parts of the films. Achieving this, as well as good reproducibility of the synthesis of the films, was possible due to the presence of Au/Si globules arranged with the same periodicity and catalyzing the growth of ZnO. Similar results, demonstrating the high homogeneity of the optical properties of the samples, were obtained in the study of isometric ZnO microcrystals, also grown on a regular system of Si whiskers [40]. A good degree of reproducibility was also reached for that microstructure. Thus, it can be argued that the use of substrates with a regular array of vertically aligned Si whiskers makes it possible to reproducibly create ZnO structures with a high degree of uniformity of optical properties.

## 5. Conclusions and Outlook

In this work, the morphological and structural features, as well as luminescent properties, of free-standing ZnO/Zn composite films were studied. It was shown that such films can be obtained using silicon wafers with a regular system of microwhiskers as substrates for vapor transport synthesis. The small area of contact between the formed film and the substrate makes it easy to separate them from each other to obtain a free-standing film. The films have different structures and surface morphologies on their front and reverse sides. While the front side was characterized by the presence of large ZnO/Zn microcrystals, the reverse side of the films had polycrystalline structures and a highly developed surface morphology. The back polycrystalline part of the films consisted of microcrystallites and nanocrystallite agglomerates, which were presumably of zinc and ZnO, respectively.

The fabricated films exhibited several interesting properties that make good prospects for their further research. Among them, one can especially highlight the following ones: (i) a high degree of flexibility with a good strength to bending, which is provided by the zinc phase; (ii) a significant difference of the visible luminescence intensity on the front and reverse sides of the films, which is due to different morphology, stoichiometry and growth conditions for the forming crystals on the different sides; and (iii) a high homogeneity of optical properties and growth reproducibility due to using a regular array of Si microwhiskers.

The highly developed surface morphology on the films’ reverse side facilitates the study of such structures towards applications in photoelectric conversion, optical-based sensors and photocatalysis, which require a high surface-to-volume ratio. Strong light trapping in such a structure is an important advantage of the films for possible use as the key elements of photovoltaic converters. It should be noted that additional studies are needed to clarify the porous properties of the films. The flexibility and high tensile strength of the films makes them useful for developing flexible devices. The results of this study, along with those obtained previously [40], indicate the possibility of using silicon substrates with a regular system of microwhiskers for the reproducible VLS synthesis of ZnO-based microstructures with a high degree of uniformity of the optical properties. In addition, by varying the growth conditions—in particular, the ratio of excess zinc and zinc reacting with oxygen—it is possible to obtain structures with different morphology and a ZnO/Zn phase ratio. The proposed technology for the formation of flexible ZnO/Zn films can be extended to other structures and materials. All of the above emphasizes the relevance of further studies of such structures, synthesized on regular arrays of Si whiskers.

## Data Availability

Not applicable.
